# Intergenerational Relation Trajectories in Multi-Child Families and Depressive Symptoms of Older Parents in China

**DOI:** 10.1093/geroni/igaf122.1751

**Published:** 2025-12-31

**Authors:** Chang Yu, Jiaowei Gong, Emma Zang

**Affiliations:** University at Buffalo, SUNY, Getzville, New York, United States; Harvard University, Cambridge, Massachusetts, United States; Yale University, New Haven, Connecticut, United States

## Abstract

**Objectives:**

While close intergenerational relationships are linked to fewer depressive symptoms in older parents, less is known about how these relationships evolve over time, how different aspects of these relationships (e.g., emotional closeness, economic and housework support, frequency of contact) function together, and how relationships with multiple children in a family shape mental health. This study examines trajectories of intergenerational relationships in multi-child families and their associations with depressive symptoms among older Chinese parents across socioeconomic (SES) groups.

**Methods:**

Using nationally representative panel data from the China Longitudinal Aging Social Survey (2014–2020, N = 1,637), we applied group-based trajectory modeling to track changes in intergenerational relationships over six years, considering overall, best, and worst child-parent relationships within families. Linear regressions assessed how these relationship trajectories relate to depressive symptoms among adults aged 60+, with attention to differences by SES (education, income, hukou).

**Results:**

Across average, best, and worst relationships, we identified four trajectories: persistently strained, slight decline with sustained positivity, steadily strengthening, and rapidly strengthening. Compared to persistently strained relationships, steadily and rapidly strengthening relationships were associated with fewer depressive symptoms, particularly for low-SES parents. The worst relationship trajectory best predicted depressive symptoms, while the average trajectory lost significance when controlling for best and worst relationships.

**Discussion:**

Findings underscore the mental health impact of relationship dynamics, especially for low-SES parents, highlighting the need for government-supported caregiving programs in China.

